# Dihydroartemisinin Enhances Apo2L/TRAIL-Mediated Apoptosis in Pancreatic Cancer Cells via ROS-Mediated Up-Regulation of Death Receptor 5

**DOI:** 10.1371/journal.pone.0037222

**Published:** 2012-05-30

**Authors:** Rui Kong, Guang Jia, Zhuo-xin Cheng, Yong-wei Wang, Ming Mu, Shuang-jia Wang, Shang-ha Pan, Yue Gao, Hong-chi Jiang, De-li Dong, Bei Sun

**Affiliations:** 1 Department of Pancreatic and Biliary Surgery, The First Affiliated Hospital of Harbin Medical University, Harbin, People's Republic of China; 2 Department of Biology, School of Agriculture and Life Science, University of Wisconsin-Madison, Madison, Wisconsin, United States of America; 3 Department of Pharmacology, Harbin Medical University, Harbin, China; Bauer Research Foundation, United States of America

## Abstract

**Background:**

Dihydroartemisinin (DHA), a semi-synthetic derivative of artemisinin, has recently shown antitumor activity in various cancer cells. Apo2 ligand or tumor necrosis factor-related apoptosis-inducing ligand (Apo2L/TRAIL) is regarded as a promising anticancer agent, but chemoresistance affects its efficacy as a treatment strategy. Apoptosis induced by the combination of DHA and Apo2L/TRAIL has not been well documented, and the mechanisms involved remain unclear.

**Methodology/Principal Findings:**

Here, we report that DHA enhances the efficacy of Apo2L/TRAIL for the treatment of pancreatic cancer. We found that combined therapy using DHA and Apo2L/TRAIL significantly enhanced apoptosis in BxPC-3 and PANC-1 cells compared with single-agent treatment *in vitro*. The effect of DHA was mediated through the generation of reactive oxygen species, the induction of death receptor 5 (DR5) and the modulation of apoptosis-related proteins. However, N-acetyl cysteine significantly reduced the enhanced apoptosis observed with the combination of DHA and Apo2L/TRAIL. In addition, knockdown of DR5 by small interfering RNA also significantly reduced the amount of apoptosis induced by DHA and Apo2L/TRAIL.

**Conclusions/Significance:**

These results suggest that DHA enhances Apo2L/TRAIL-mediated apoptosis in human pancreatic cancer cells through reactive oxygen species-mediated up-regulation of DR5.

## Introduction

Pancreatic cancer is one of the most lethal digestive system malignancies and has a very poor prognosis [1], [2]. Conventional chemotherapy has shown limited survival benefit when combined with surgical resection [3]. Thus, an effective treatment strategy for pancreatic cancer is urgently needed.

Artemisinin (the active principle of the Chinese medicinal herb, Artemisia annua [4], [5]) and its derivatives are extremely effective antimalarial drugs with few adverse side effects [6]. Dihydroartemisinin (DHA), a derivative of artemisinin, is a more water-soluble and effective antimalarial than artemisinin [7]. Recently, it has been demonstrated that DHA effectively kills various types of cancer cells [8]–[13]. Furthermore, we have determined that DHA can significantly inhibit cell growth and induce the apoptosis of pancreatic cancer cells in a dose- and time-dependent manner but is essentially non-toxic to normal cells [9]. In previous studies, Agtmael [14] and O'Neill PM [5] demonstrated that artemisinin contains an endoperoxide bridge, which is essential for the reaction with iron to form reactive oxygen species (ROS). Moreover, it has been demonstrated that cancer cells take up increased amounts of iron to produce higher levels of intracellular ROS than are found in normal cells [15]. Thus, it is likely that the potency of DHA against cancer cells is related to the generation of ROS [16], but the actual mechanism of DHA action is poorly understood.

Apo2 ligand or tumor necrosis factor-related apoptosis-inducing ligand (Apo2L/TRAIL) is a member of the TNF superfamily, which serves as an effective anticancer agent due to its cancer cell specificity and potent antitumor activity. Apo2L/TRAIL induces apoptosis in cancer cells through interactions with death receptor 4 (DR4) and DR5, which form the death-inducing signal complex (DISC) by binding to FADD and caspase-8 or caspase-10 [17]–[19]. The DISC then in turn initiates a protease cascade, which activates downstream effector caspases, including caspase-3. In addition, many apoptosis-related proteins downstream of the death receptors, such as caspase-6, caspase-7 and caspase-9, participate in Apo2L/TRAIL-induced apoptosis [20]. A number of studies have shown that terpenoids can up-regulate the expression of DR4 and/or DR5 in human cancer cells [12], [20], [21]. Thus, agents that can up-regulate Apo2L/TRAIL receptors and down-regulate anti-apoptotic proteins have the potential to enhance the apoptotic effects of Apo2L/TRAIL. In this study, we sought to determine the effect of DHA on Apo2L/TRAIL-induced apoptosis in pancreatic cancer cells. Our results show that DHA can potentiate the apoptotic effects of Apo2L/TRAIL through up-regulation of DR5 expression in human pancreatic cancer cells.

## Results

### DHA and Apo2L/TRAIL synergistically inhibit the growth of pancreatic cancer cells

**Figure 1:**
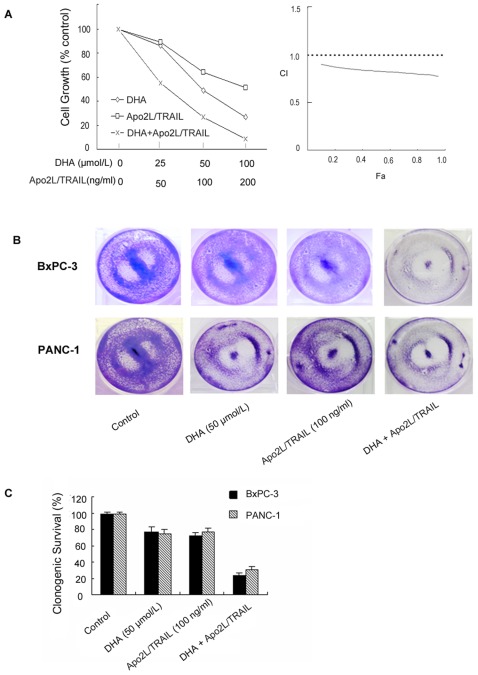
DHA synergistically enhances Apo2L/TRAIL-induced cell death in BxPC-3 cells. (A) Cells were treated with DHA alone, Apo2L/TRAIL alone or a combination of the two agents and incubated at 37°C for 72 h. The viability of the cells was assessed using the MTT method. Combination index (CI) versus fraction affected (Fa) plots obtained from median-effect analysis of Chou-Talalay. A CI>1 indicates antagonism,  = 1 indicates additivity, and <1 indicates synergy. (B) The clonogenic assay was performed as described in the Materials and Methods section. The cells were treated with DHA (50 µmol/L) alone, Apo2L/TRAIL (100 ng/ml) alone or the combination of the two drugs for 24 h and washed with PBS. The cells were then incubated for an additional 7 d and stained with crystal violet. (C) Clonogenic survival is presented as the percentage of surviving colonies formed in drug-treated cells with respect to untreated cells.

To determine whether DHA and Apo2L/TRAIL act synergistically to inhibit the growth of pancreatic cancer cells, we measured cell viability in BxPC-3 and PANC-1 cell lines in response to different treatment regimens. Dose-dependent growth inhibition was observed when BxPC-3 cells were treated with either DHA (0–100 µmol/L) or Apo2L/TRAIL (0–200 ng/ml) alone (Figure 1A left). However, treatment with higher doses of DHA and Apo2L/TRAIL in combination (50 µmol/L and 100 ng/ml, respectively and 6 µmol/L and 200 ng/ml, respectively) resulted in more potent growth inhibition than treatment with either compound alone (Figure 1A left). To more accurately determine the effects of combination therapy, data were examined using median effect analysis to determine the type of interactions which occurred, i.e. antagonism (CI>1), additivity (CI = 1) or synergism (CI<1). The synergistic effect (CI<1) was observed in the combination therapy (Figure 1A right). Similarly, a suggestive synergistic effect of DHA and Apo2L/TRAIL on cell growth was observed in PANC-1 cells (data not shown).

Next, we performed a clonogenic assay to determine whether DHA enhances the effect of Apo2L/TRAIL on long-term colony formation. The cells were treated with DHA (50 µmol/L) and Apo2L/TRAIL (100 ng/ml) either alone or in combination for 24 h. The cells were then washed with PBS, incubated in fresh medium and, seven days later, were stained with crystal violet. The clonogenic assay indicated that the number of surviving colonies in the group that received the combination of DHA and Apo2L/TRAIL was dramatically lower than in the groups treated with DHA or Apo2L/TRAIL alone. These results indicate that the combined treatment with DHA and Apo2L/TRAIL significantly reduced the number of reproductive cells (Figure 1B and 1C).

### DHA enhances Apo2L/TRAIL-induced apoptosis dependent on reactive oxygen species

To determine whether the ability of DHA to enhance the cytotoxicity of Apo2L/TRAIL was mediated by ROS-induced apoptosis, we used flow cytometry and laser scanning confocal microscopy to measure the rate of apoptosis and intracellular ROS in BxPC-3 and PANC-1 cells.

**Figure 2:**
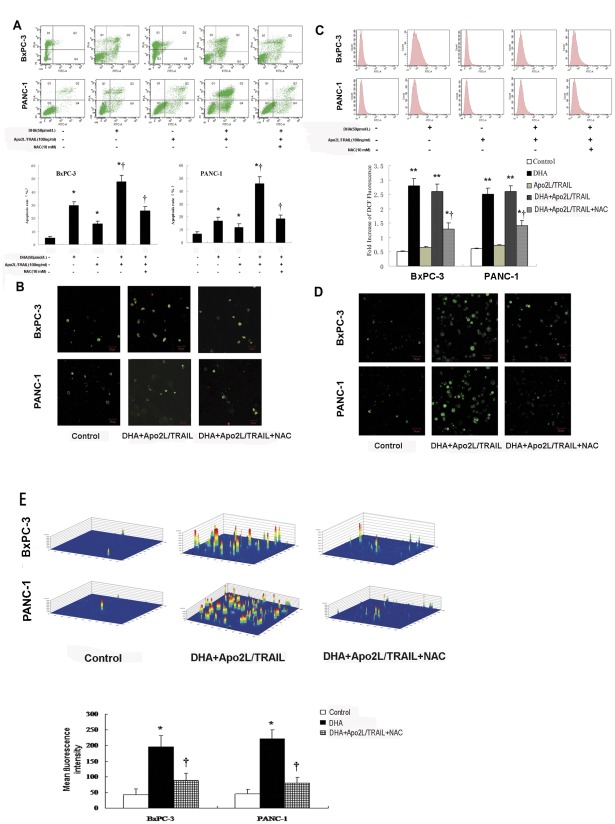
DHA-induced apoptosis is caused by ROS. (A) Apoptosis of pancreatic cancer cells. BxPC-3 and PANC-1 cells were treated with DHA (50 µmol/L) and Apo2L/TRAIL (100 ng/ml) as indicated. Flow cytometry was performed to measure apoptosis rates (%). A significant increase in the apoptosis rate compared with the control is denoted by “*” (*P*<0.05), a significant increase compared with DHA- or Apo2L/TRAIL-treated cells is denoted by “†” (*P*<0.01), and a significant decrease compared with DHA+Apo2L/TRAIL-treated cells is denoted by “‡” (*P*<0.01). Representative histograms from cytometrically analyzed BxPC-3 and PANC-1 cells treated with control, DHA, Apo2L/TRAIL, DHA+Apo2L/TRAIL or NAC (10 mM). (B) Laser scanning confocal microscopy of cells. Representative photographs were taken of the control BxPC-3 and PANC-1 cells and of BxPC-3 and PANC-1 cells treated with DHA+Apo2L/TRAIL and DHA+Apo2L/TRAIL+NAC. (C) Levels of intracellular ROS measured in vitro. BxPC-3 and PANC-1 cells were treated with DHA (50 µmol/L), Apo2L/TRAIL (100 ng/ml), DHA+Apo2L/TRAIL, or pretreated with NAC (10 mM) and then treated with DHA+Apo2L/TRAIL for 6 h. Untreated cells served as the control. The cells were incubated with DCFHDA and then subjected to flow cytometry to measure the levels of intracellular ROS, as represented by DCF fluorescence. A significant increase in DCF fluorescence compared with the control is denoted by “*” (*P*<0.05), a highly significant difference compared with the control is denoted by “**” (*P*<0.01), and a significant reduction compared with the DHA+Apo2L/TRAIL treatment is denoted by “†” (*P*<0.05). (D) Representative photographs are shown for DCFHDA-stained cells observed using laser scanning confocal microscopy. The green fluorescence represents intracellular ROS. (E) The mean fluorescence intensity was measured for the DCFHDA-stained cells, and the respective 3-dimensional horizontal plane images were produced by laser scanning confocal microscopy. A significant difference from the control is denoted by “*” (*P*<0.01), and a significant difference from the DHA+Apo2L/TRAIL treatment is denoted by “†” (*P*<0.05).

BxPC-3 and PANC-1 cells were treated with DHA (50 µmol/L) and/or Apo2L/TRAIL (100 ng/ml). Following 72 h of treatment, the cells were stained with Annexin V and propidium iodide (PI), subjected to flow cytometry and viewed by laser scanning confocal microscopy to measure the rate of apoptosis. Representative histograms from the flow cytometry experiments indicate that the rates of apoptosis of control BxPC-3 cells and cells treated with DHA, Apo2L/TRAIL or the combination of both were 5.1%, 29.4%, 15.5% and 47.8%, respectively (Figure 2A). With respect to the PANC-1 cells, the rates of apoptosis were 6.7%, 18.6%, 11.9% and 45.8%, respectively. Thus, treatment with DHA or Apo2L/TRAIL alone significantly increased the rate of apoptosis of BxPC-3 cells compared with the controls (both *P*<0.05). Furthermore, treatment with the combination of DHA and Apo2L/TRAIL led to a further increase in the rate of apoptosis compared with treatment with DHA or Apo2L/TRAIL alone (both *P*<0.01). Similar results were obtained with PANC-1 cells. The extent of apoptosis, as detected by Annexin V labeling, approximates the reduction in cell viability following treatment with DHA, Apo2L/TRAIL or the combination of both, suggesting that apoptosis accounts for most of the observed cytotoxicity. However, pretreatment with N-acetyl cysteine (NAC, 10 mM) was able to significantly reduce the amount of apoptosis caused by the combined treatment of DHA and Apo2L/TRAIL in BxPC-3 and PANC-1 cells (both *P*<0.01). Taken together, these data indicate that ROS mediates the ability of DHA to enhance Apo2L/TRAIL-induced apoptosis. The use of laser scanning confocal microscopy confirmed the increased apoptosis measured by flow cytometry, as shown in the representative photographs (Figure 2B). BxPC-3 and PANC-1 cells treated with DHA and Apo2L/TRAIL displayed more numerous early- and late-stage apoptotic cells than the controls, which had very few early-stage apoptotic cells.

BxPC-3 and PANC-1 cells were incubated with DHA (50 µmol/L), Apo2L/TRAIL (100 ng/ml) or the combination of both for 12 h, and DCF fluorescence was recorded as a measure of intracellular ROS levels. As shown in Figure 2C, the levels of intracellular ROS were significantly (*P*<0.01) higher in cells that had been treated with DHA and the combination of DHA and Apo2L/TRAIL than in control cells. However, Apo2L/TRAIL treatment alone had little effect on intracellular ROS levels in BxPC-3 and PANC-1 cells. To confirm that ROS generation is responsible for enhancing Apo2L/TRAIL-induced apoptosis, the cells were pretreated with 10 mM NAC, a well-known inhibitor of ROS production, for 6 h and then incubated with the combination of DHA and Apo2L/TRAIL. Laser scanning results indicate that pretreatment with NAC significantly (*P*<0.05) reduced the level of intracellular ROS in all cell types compared with the cells treated with DHA and Apo2L/TRAIL alone. Confocal microscopy in combination with DCFHDA staining, where green fluorescence represents the intracellular ROS (Figure 2D), revealed that intracellular ROS was significantly (*P*<0.01) higher in DHA-treated cells than in control cells. However, pretreatment with NAC significantly (*P*<0.05) reduced the mean fluorescence intensity in the DHA- and Apo2L/TRAIL-treated cells (Figure 2E).

### DHA regulates apoptosis-related gene expression

**Figure 3:**
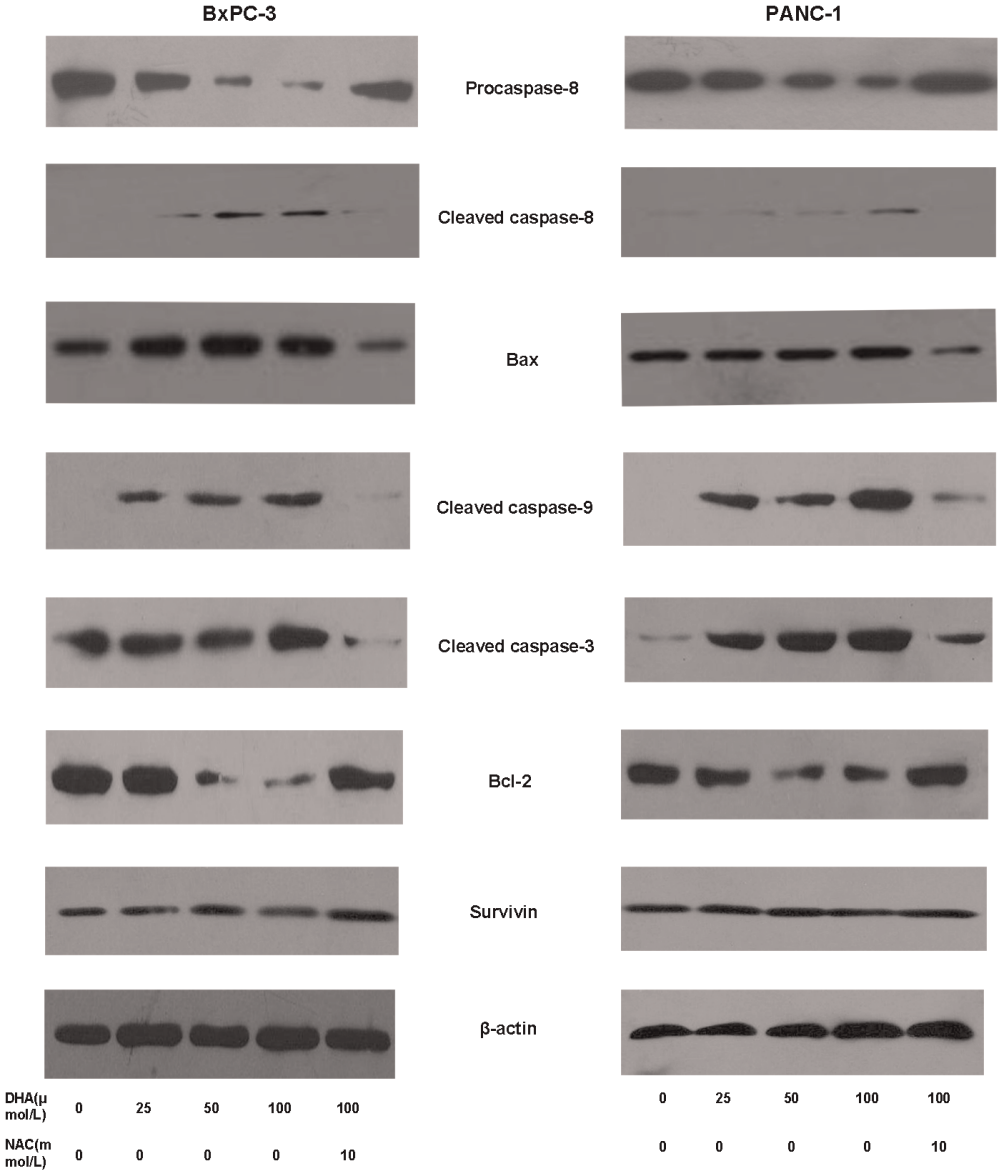
The expression of apoptosis-related genes. BxPC-3 and PANC-1 cells were treated with various concentrations of DHA (0, 25, 50, 100 µmol/L) and pretreated with NAC followed by DHA (100 µmol/L) for 72 h. Whole cell extracts were prepared and analyzed by western blotting using antibodies against Bcl-2, Bax, surviving, caspase-3, caspase-8, and caspase-9. DHA significantly up-regulated the expression of Bax, caspase-3, caspase-8 and caspase-9, and down-regulated the expression of Bcl-2. However, DHA had little influence on the expression of survivin. DHA with NAC (10 mM) pretreatment did not up-regulate the expression of caspase-8, Bax, caspase-9, and caspase-3. β-actin served as an internal control.

Next, we analyzed the expression of apoptosis-related genes mediated by ROS after treatment with DHA. BxPC-3 and PANC-1 cells were treated with various concentrations of DHA (0, 25, 50 and 100 µmol/L) or pretreated with NAC (10 mM) followed by the addition of DHA (100 µmol/L) for 72 h, and the expression levels of Bcl-2, Bax, surviving, caspase-3, caspase-8 and caspase-9 were detected by western blot analysis. In response to DHA, the expression levels of Bax, caspase-3, caspase-8 and caspase-9 were significantly up-regulated, whereas Bcl-2 expression decreased in a dose-dependent manner, and the expression of survivin remained unchanged in the BxPC-3 and PANC-1 cells. Interestingly, pretreatment with NAC (10 mM) blocked the ability of DHA to up-regulate the expression of caspase-8, Bax, caspase-9 and caspase-3. It was also found that DHA significantly enhanced Apo2L/TRAIL-mediated apoptosis in both cell lines, and this correlated with increased activation of caspase-8, Bax, caspase-9 and caspase-3 (Figure 3).

### DHA-induced up-regulation of DR5 is dependent on reactive oxygen species

**Figure 4:**
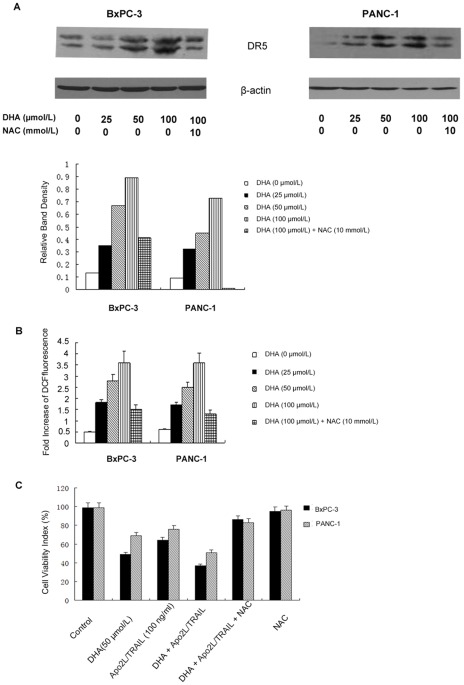
Up-regulation of DR5 by DHA was mediated by ROS. (A) BxPC-3 and PANC-1 cells were treated with the indicated doses of DHA for 48 h. Whole cell extracts were prepared and analyzed for DR5 expression using western blotting. DHA had dose-dependent effects on the expression of DR5. The DHA-induced increase in DR5 levels was significantly blocked by pretreatment with 10 mM NAC. β-actin served as an internal control. (B) The cells were collected and analyzed using flow cytometry. A gradual increase in fluorescence was observed in cells treated with 25, 50 and 100 µmol/L DHA, respectively, indicating a dose-dependent increase in the production of ROS in response to DHA treatment in the two cell lines. The production of ROS was markedly inhibited by pretreating the cells with NAC (10 mM). (C) The cells were treated with DHA (50 µmol/L) alone, Apo2L/TRAIL (100 ng/ml) alone or a combination of the two agents. The cells were also treated with NAC (10 mM) alone or pretreated with NAC and incubated at 37°C for 72 h. The viability of the cells was assessed using the MTT method and the viability index (%) was calculated. Significant differences are denoted by “*” (*P*<0.01).

We next examined whether ROS was involved in DHA-induced DR5 expression. BxPC-3 and PANC-1 cells were treated with various concentrations of DHA for 48 h, whole cell extracts were prepared and the expression of DR5 protein was examined. We found that DHA had dose-dependent effects on the expression of DR5 (Figure 4A). Thus, we sought to determine whether the generation of ROS is directly associated with the ability of DHA to induce DR5 expression. FACS detection showed that treatment of BxPC-3 and PANC-1 with DHA increased intracellular ROS levels in a dose-dependent manner, and the DHA-induced increase in ROS levels was significantly blocked by pretreatment with 10 mM NAC (Figure 4B). Importantly, pretreatment with NAC markedly inhibited the DHA-induced up-regulation of DR5 expression (Figure 4A).

We next examined whether scavenging of ROS could attenuate the Apo2L/TRAIL-induced cell death enhanced by DHA. As shown in Figure 4C, DHA markedly enhanced Apo2L/TRAIL-induced cell death in BxPC-3 and PANC-1 cells. However, pretreatment with NAC significantly reduced the ability of DHA to induce cell death from 63% to 14% in BxPC-3 cells and from 31% to 17% in PANC-1 cells. Taken together, these data indicate that the ROS-mediated up-regulation of DR5 is critical for the observed DHA enhancement of Apo2L/TRAIL-induced apoptosis.

### Apo2L/TRAIL-induced apoptosis is mediated by DHA-regulated DR5 expression

**Figure 5:**
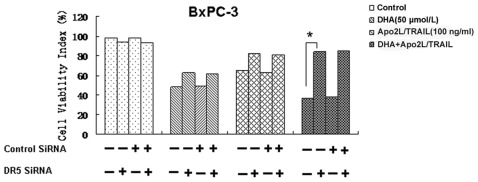
Effects of knockdown of DR5 expression on DHA-induced cytotoxicity and cell apoptosis of Apo2L/TRAIL. BxPC-3 and PANC-1 cells were transfected with DR5 siRNA and control siRNA, either alone or in combination. After 48 h, the cells were treated with 50 µmol/L DHA for 24 h, and whole cell extracts were subjected to western blotting to test for the expression of DR5. Transfection of cells with siRNA targeting DR5 specifically silenced the expression of DR5. The cells were seeded on a chamber slide and transfected with siRNAs. After 48 h, the cells were treated with 50 µmol/L DHA, 100 ng/mL Apo2L/TRAIL, either alone or in combination, and incubated at 37°C for 72 h. The viability of the cells was assessed using the MTT method, and the viability index (%) was calculated. Silencing of DR5 by siRNA reduced the cytotoxic effect of the combination of DHA and Apo2L/TRAIL but not of DHA alone. Significant differences are denoted by “*” (*P*<0.01).

We also investigated whether DHA-mediated DR5 expression is required for Apo2L/TRAIL-induced apoptosis in pancreatic cancer cells. To determine the role of DR5 in Apo2L/TRAIL-induced apoptosis, we used siRNAs to down-regulate DR5 expression. We next examined whether the suppression of DR5 by siRNA could abrogate the sensitizing effects of DHA on Apo2L/TRAIL-induced cell death. Transfection of cells with DR5 siRNA increased the viability of BxPC-3 cells by 47% (*P*<0.01) and the viability of PANC-1 cells by 42% (data not shown), compared with the combination treatment alone (Figure 5); treatment with control siRNA had no effect (Figure 5). These results reveal, therefore, that the effect of DHA on Apo2L/TRAIL-induced apoptosis had been effectively abolished in the cells that were transfected with DR5 siRNA, indicating that DR5 is a major mediator of Apo2L/TRAIL-induced apoptosis.

### DHA and Apo2L/TRAIL significantly inhibit the growth of human pancreatic cancer cells *in vivo*

**Figure 6:**
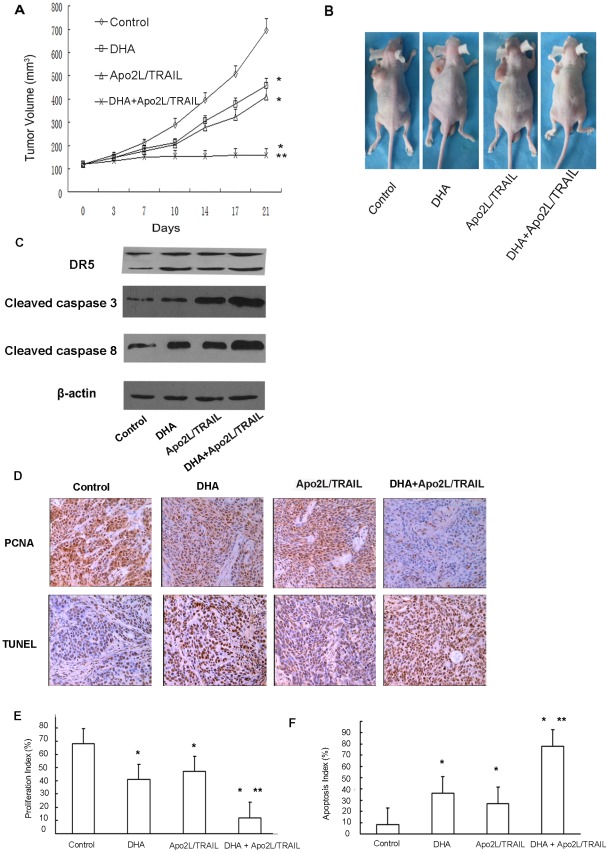
Tumor growth, tumor gene expression, tumor proliferation and apoptosis *in vivo*. (A) BxPC-3 tumors were established subcutaneously in mice. When the tumors reached approximately 120 mm^3^ in volume, the mice were randomly assigned to control, DHA, Apo2L/TRAIL, or DHA+Apo2L/TRAIL groups and treated as described in the methods section. The sizes (measured in mm^3^) of the tumors were monitored and recorded. A significant difference in tumor volume from the control is denoted by “*” (*P*<0.05), and a significant reduction compared to the DHA or Apo2L/TRAIL-treated tumors is denoted by “**” (*P*<0.01). (B) Representative animals and tumors are shown for each group. (C) Tumors from control mice and from mice treated with DHA, Apo2L/TRAIL, and DHA+Apo2L/TRAIL were homogenized and subjected to western blot analysis to detect the expression of caspase-3 and caspase-8. β-actin served as an internal control. (D) Analysis of proliferation marker PCNA by immunohistochemistry and apoptotic status of tumor cells by in situ TUNEL assay. PCNA positive (E) and TUNEL-positive (F) cells were also counted under microscope to calculate the proliferation index and apoptotic index, respectively. “*”: *P*<0.05, compared with control. “**”: *P*<0.01, compared with single agent.

To investigate the effect of DHA and/or Apo2L/TRAIL on cell growth *in vivo*, BxPC-3 cells were subcutaneously xenografted in nude mice. Neither the DHA nor the Apo2L/TRAIL groups demonstrated a significant reduction in tumor volume compared with the control group (*P*<0.05). However, mice treated with the combination of DHA and Apo2L/TRAIL had tumors that were not only significantly smaller than the control (*P*<0.01), but were also smaller than those treated with either agent alone (*P*<0.05), demonstrating higher suppression on tumor growth *in vivo* (Figure 6A and B). Western blot analysis of tumor tissue lysates also revealed that the expression levels of DR5, caspase-3 and caspase-8 were significantly up-regulated in the group treated with the combination therapy and to a higher extent than the groups treated with either DHA or Apo2L/TRAIL alone (Figure 6C). These results are consistent with our *in vitro* studies, providing further evidence that DHA can potentiate the antitumor activity of Apo2L/TRAIL *in vivo*.

Cell proliferation marker PCNA and cell apoptosis were further examined *in situ* in tumor samples from the four groups. As shown in Figure 6D and E, DHA or Apo2L/TRAIL treated groups had reduced expression of PCNA compared to control group (*P*<0.05). While mice treated with the combination of DHA and Apo2L/TRAIL had expression of PCNA that was not only lower than the control (*P*<0.01), but was also lower than either agent treated alone (*P*<0.05). Mono-therapy with either DHA or Apo2L/TRAIL produced significantly higher apoptosis than control (*P*<0.05), while combined therapy produced even more apoptosis not only than the control (*P*<0.01), but also than groups treated with single agent (*P*<0.05, Figure 6D and F).

## Discussion

In the present study, we show that DHA, a semisynthetic derivative of artemisinin, can enhance the apoptotic effect of Apo2L/TRAIL against pancreatic cancer cells. DHA acts synergistically with Apo2L/TRAIL to inhibit proliferation and induce apoptosis through the ROS-mediated up-regulation of DR5 in both BxPC-3 and PANC-1 cells. Furthermore, we demonstrated that DHA can sensitize pancreatic cancer cells to Apo2L/TRAIL treatment *in vivo*.

Soluble Apo2L/TRAIL, which is emerging as an attractive anticancer agent, has been evaluated in several clinical trials. However, many reports indicate that the majority of pancreatic carcinoma cell lines are resistant to Apo2L/TRAIL [22]–[25]. Cancer cell resistance to Apo2L/TRAIL is one of the greatest barriers to the further exploitation of this therapy. Thus, agents that can either potentiate the effect of Apo2L/TRAIL or overcome resistance are urgently needed [26]. The goals of this study were to determine whether combining DHA with Apo2L/TRAIL could promote synergistic antitumor activity and to understand the exact mechanism by which combination therapy using these agents elicits pancreatic cancer cell death *in vitro* and *in vivo*.

Although many neoplasms display some resistance to Apo2L/TRAIL in clinical trials, a growing body of data demonstrates that Apo2L/TRAIL can selectively kill cancer cells without harming normal cells, which could alleviate many side effects of conventional chemotherapy. Notably, many reports have shown that some plant extracts and chemotherapeutic agents can sensitize cancer cells to Apo2L/TRAIL-induced cell death [17], [26]–[29], indicating that Apo2L/TRAIL resistance can be overcome with the use of effective sensitizers. Recently, DHA has been shown to produce strong antitumor effects either alone or in combination with chemotherapeutic agents with minimal host toxicity against various kinds of carcinoma *in vitro* and *in vivo* [8]–[10], [30]–[32]. Meanwhile, the effect of DHA against pancreatic cancer has been confirmed in our previous studies [9], [30]. However, it is unknown whether DHA and Apo2L/TRAIL act synergistically against pancreatic cancer. In the present study, we found that the combination of DHA and Apo2L/TRAIL resulted in significantly greater growth inhibition of BxPC-3 and PANC-1 cells *in vitro* than when either agent was used alone. Our results indicate that DHA can synergistically enhance Apo2L/TRAIL-mediated cytotoxicity and sensitize human pancreatic cancer cell lines to Apo2L/TRAIL-induced apoptosis.

Several reports have shown that ROS generation is involved in Apo2L/TRAIL-mediated apoptosis [33], [34]. ROS is also generally accepted to be associated with tumorigenesis and metastasis, although this correlation is often complex and contradictory [35]. In fact, most anti-neoplastic agents have well-established mechanisms of action that involve the generation of ROS [36]. In cancer cells, drug-induced oxidative stress superimposes on intrinsic oxidative stress, resulting in a potent ROS-mediated cytotoxic response that preferentially kills tumor cells or inhibits their proliferation [37]. An endoperoxide bridge in artemisinin reacts with intracellular iron to generate free radicals, which can lead to macromolecular damage and cell death [38], [39]. DHA, a derivative of artemisinin, has been shown to cause the death of human papillomavirus-expressing cell lines by inducing oxidative stress that leads to the generation of ROS [8]. Recent work has also demonstrated that cultured human metastatic melanoma cells are sensitive to DHA-induced apoptosis and exhibit up-regulation of cellular oxidative stress, phosphatidylserine externalization and procaspase-3 cleavage [40]. In our study, we found that the levels of intracellular ROS significantly increased by DHA treatment in the presence and absence of Apo2L/TRAIL, whereas treatment with Apo2L/TRAIL alone had little effect on intracellular ROS in BxPC-3 and PANC-1 cells. We also found that the ability of DHA to enhance Apo2L/TRAIL-induced apoptosis could be attenuated by pretreatment with NAC. Therefore, our results provide a novel mechanism by which DHA can act synergistically with Apo2L/TRAIL to exert their combined pro-apoptotic effects in pancreatic cancer, at least in part through ROS generation. In colon cancer cells, apoptosis induced by Apo2L/TRAIL alone is also regulated by the generation of ROS [41].

Two primary pathways are known to initiate cellular apoptosis: the death receptor-induced, extrinsic pathway and the intrinsic, mitochondrial pathway [42], [43]. We previously reported that DHA could trigger the mitochondrial pathway to induce apoptosis by regulating the expression of Bcl-2 and Bax, leading to the release of cytochrome c from the mitochondria and activating the downstream initiator caspase-9, resulting in the activation of the effector caspases and the induction of apoptosis [44]. However, several studies have illustrated that Apo2L/TRAIL resistance in cancer cell lines involves the expression of the anti-apoptotic protein c-FLIP and down-regulation of the pro-apoptotic protein caspase-8 [20], [27], [45]. Some chemotherapeutic agents, including zerumbone [20] and garcinol [27], can significantly down-regulate the expression of c-FLIP and/or up-regulate the expression of caspase-8 through ROS to increase the sensitivity of cells to Apo2L/TRAIL. It is recognized that caspase-8 is a direct downstream target of DR5, and pro-caspase-8 recruits DISC to activate caspase-8, resulting in activation of downstream effector caspases [46]. In our study, DHA up-regulates caspase-8 expression in a dose-dependent manner to initiate cellular apoptosis. We also show that DHA modulates the expression of DR5 in a dose-dependent manner and that the increased expression of DR5 is linked to the observed changes in oxidative stress. Our data indicate that ROS is one of the upstream regulators responsible for the DHA-mediated induction of DR5 and that DHA can potentiate Apo2L/TRAIL-induced cell apoptosis through the extrinsic death-receptor pathways. These results are consistent with those reported in a previous study [20]. The crosstalk between these two pathways is likely due to caspase-8-mediated cleavage of Bid and its subsequent translocation to the mitochondria where it initiates the intrinsic apoptosis pathway [28]. Taken together, these results suggest that, in addition to the mitochondrial-mediated apoptotic pathways, DHA-dependent ROS generation also plays a pivotal role in the synergistic enhancement of Apo2L/TRAIL-induced apoptosis via the extrinsic death-receptor pathways in pancreatic cancer cells.

Here, we successfully used the ROS scavenger, NAC, to inhibit oxidative stress. We also found that NAC abolished the ability of DHA to induce DR5 expression, which is consistent with previous reports regarding cancer chemopreventive agents, including DHA [12], curcumin [47], sulforaphane [48] and zerumbone [20]. To further investigate whether down-regulation of DR5 could attenuate Apo2L/TRAIL chemosensitivity, we utilized siRNAs to deplete cells of DR5. We found that DR5 siRNA significantly reduced the expression of DR5 in BxPC-3 and PANC-1 cells. The depletion of DR5 by siRNA significantly attenuated apoptosis in response to the combination therapy, indicating that the ability of DHA to augment Apo2L/TRAIL-induced apoptosis is mediated by up-regulation of DR5. These results are similar to those found in previous reports which demonstrate that down-regulation of DR5 expression efficiently reduces the sensitization to Apo2L/TRAIL-induced apoptosis by tunicamycin [49]. Taken together, these findings show that DHA can enhance Apo2L/TRAIL-mediated apoptosis through ROS-mediated up-regulation of DR5.

Using xenograft models, we found that the volume of tumors in mice treated with the combination of DHA and Apo2L/TRAIL remained essentially unchanged over the course of our study, whereas tumor volume increased considerably in the control group and, to a lesser extent, in the groups treated with either DHA or Apo2L/TRAIL alone. These results are consistent with those of our *in vitro* studies, demonstrating that DHA potentiates the antitumor activity of Apo2L/TRAIL.

In summary, we conclude that DHA can potentiate Apo2L/TRAIL-mediated apoptosis in pancreatic cancer cells through ROS-mediated up-regulation of DR5. Considering that DHA is not toxic to normal cells and that Apo2L/TRAIL is selective to tumor cells, we predict that the use of a combination of DHA with Apo2L/TRAIL could be a safe and effective treatment compared with conventional chemotherapy. These findings have implications for the use of Apo2L/TRAIL as a cancer therapy, especially for tumors that develop resistance to Apo2L/TRAIL. Importantly, xenograft studies confirmed that DHA potentiates the antitumor activity of Apo2L/TRAIL *in vivo*. Thus, these results indicate that treatment with the combination of DHA and Apo2L/TRAIL may support a novel therapeutic strategy against pancreatic cancer in clinical settings.

## Materials and Methods

### Reagents, cell lines and cell culture

Human pancreatic cancer cell lines BxPC-3 and PANC-1 were obtained from the Institute of Biochemistry and Cell Biology at the Chinese Academy of Sciences, Shanghai, China. The cells were routinely cultured at 37°C in RPMI 1640 medium supplemented with 10% fetal calf serum, penicillin (100 U/ml) and streptomycin (100 µg/ml) in a CO_2_ incubator. All cell lines were routinely screened for mycoplasma contamination, using the Mycoplasma Stain Assay Kit (Beyotime Institute of Biotechnology, Beijing, China). The antibodies (ABs) against caspase-9, Bcl-2, caspase-3, Bax, surviving, PCNA and β-actin were purchased from Santa Cruz Biotechnology, California, USA. The anti-caspase-8 AB was purchased from Cell Signaling Inc., Massachusetts, USA. The anti-DR5 AB was obtained from Biosynthesis Biotechnology Co. Ltd., Beijing, China. Annexin V-FITC and propidium iodide (PI) were purchased from BaoSai Biotechnology Co. Ltd., Beijing, China. Soluble recombinant human Apo2L/TRAIL was purchased from PeproTech, USA. DHA was obtained from Sigma-Aldrich, St. Louis, Missouri, USA. Working solutions were prepared by dissolving the compound in dimethyl sulfoxide (DMSO). The final concentration of DMSO was less than 1% in all experiments.

### Cell viability assay

Cell viability was measured using MTT and crystal violet assays. BxPC-3 (4×10^3^) and PANC-1 (3×10^3^) cells were seeded onto 96-well plates. After overnight culture, the cells were treated with various concentrations of DHA, Apo2L/TRAIL or a combination of the two compounds for 72 h. Control cells were cultured in RPMI 1640 medium. After treatment, the medium was changed from a complete culture medium to a serum- and pH indicator-free medium, then the cell lines were incubated with MTT (3-(4,5-dimethylthiazol-2-yl)-2,5-diphenyl tetrazolium bromide) at 37°C for 4 h. The medium was discarded, and DMSO was added to each well at room temperature for 20 min, the absorbance was measured at 490 nm on a plate reader and the optical density (OD) was recorded. The highest concentration of Apo2L/TRAIL and DHA as single agents and in combination do not interfere with the MTT assay reagents, in the absence of cells (data not shown). The growth index (%) was calculated according to the formula: experimental OD value/control OD value ×100. All experiments were repeated in triplicate.

### Clonogenic assay

BxPC-3 and PANC-1 (3×10^4^/well) cells were seeded onto 6-well plates and cultured overnight. The medium was then replaced with complete culture medium containing various concentrations of DHA, Apo2L/TRAIL or a combination of the two compounds for an additional 24 h. Following drug exposure, the medium was replaced with fresh medium, and all cultures were incubated for an additional 7 days. The cells were then washed twice with pre-warmed PBS, and the remaining cells were stained for 2 h with a crystal violet solution (0.5% crystal violet, 20% methanol). After removal of the crystal violet solution, the plates were washed three times by immersion in a beaker filled with tap water. The plates were left to dry at 37°C, and the images were analyzed as described previously [50].

### Calculation of the combination index (CI)

After concentration-effect curves were generated for each agent, data were analyzed using the median-effect principle, which uses the method of Chou and Talalay [51], to determine whether combination treatment yields greater effects than expected from summation alone. A CI>1 indicates antagonism,  = 1 indicates additivity, and <1 indicates synergy [52].

### Annexin V and propidium iodide staining

The cells were treated as described above for 48 h, harvested, washed with PBS and the numbers of cells were counted. The cells (1×10^5^) were suspended in 100 µl of binding buffer and incubated with 5 µl of Annexin V and 5 µl of PI for 15 min at room temperature in the dark. The cell apoptosis rate (%) was measured using a Beckman Coulter Epics Altra II cytometer (Beckman Coulter, California, USA). A laser scanning confocal microscope (LSM-510, Carl Zeiss Jena GmbH, Jena, Germany) was used to visualize cells that had undergone apoptosis.

### Measurement of ROS generation

This assay is based on the incorporation of 2′,7′-dichlorofluorescein (DCF) diacetate into the cell. H_2_O_2_ and peroxidases are able to oxidize cleaved DCFH to DCF, which is highly fluorescent at 530 nm. The cells were plated at a density of 1×10^5^ in 6-well plates, allowed to attach overnight, and exposed to different concentrations of DHA for 4 h. The cells were then incubated with 10 M DCFHDA for 20 min at 37°C in a 5% CO_2_ incubator, washed and resuspended in PBS at 1×10^6^ cells/ml. The cells were analyzed by flow cytometry at an excitation wavelength of 514 nm, and the fluorescence intensity of DCF was measured at an emission wavelength of 525 nm. Untreated cells served as controls. The amount of intracellular ROS was expressed as the fold-increase of DCF fluorescence compared with the control. The experiments were repeated three times. The fluorescence intensity of intracellular DCF was observed using a laser scanning confocal microscope. The respective 3-dimensional horizontal plane images were produced using a laser scanning confocal microscope, based on the mean fluorescence intensity measured for the cells stained with DCFHDA.

### Small Interfering RNAs (siRNAs)

A double-stranded siRNA (DR5 siRNA) (sense, 5′-GACCCUUGUGCUCGUUGUCTT-3′, antisense 5′-GACAACGAGCACAAGGGUCTT-3′) was designed to target the DR5 gene [53]. A nonspecific control siRNA (sense 5′-UUCUCCGAACGUGUCACGUTT-3′; antisense 5′-ACGUGACACG UUCGGAGAATT-3′) was also designed. The siRNAs were produced by GenePharma Co. Ltd., Shanghai, China. BxPC-3 and PANC-1 cells were grown to approximately 40–50% confluence in 6- or 96-well plates and transfected with the siRNAs using Lipofectamine™ 2000 (Invitrogen) following the previously described method in serum-free medium without antibiotic supplementation [54]. After incubation for 6 h, the culture medium was refreshed, and the cells were cultured for an additional 72 h with Apo2L/TRAIL and/or various concentrations of DHA. Silencing of protein expression was confirmed by western blot analysis.

### Western blotting

The methodology has been described previously [30]. Briefly, 5×10^5^ cells were sonicated in RIPS buffer and homogenized, and cellular debris was removed by centrifugation. The protein contents of the cell and tumor tissue homogenates were determined, and 30 µg protein samples were resolved on 12% polyacrylamide SDS gels and electrophoretically transferred to polyvinylidene difluoride membranes. The membranes were blocked with 5% BSA, incubated first with primary antibodies, and subsequently with an alkaline phosphatase-conjugated secondary antibody. The membranes were then developed using 5-bromo-4-chloro-3-indolyl phosphate/nitro blue tetrazolium (Tiangen Biotech Co. Ltd., Beijing, China). The blots were stained with an anti-β-actin AB to confirm that each lane contained a similar amount of protein.

### *In vivo* studies

All surgical and care procedures administered to the animals were in accordance with institutional guidelines. Athymic, 6-week-old nude mice were obtained from the Animal Research Center, Dalian Medical University, China. Tumors were established by subcutaneous injection of 5×10^6^ BxPC-3 cells into the flanks of the mice. When the tumors reached sizes of approximately 120 mm^3^, the host mice were randomly assigned to four groups (6 in each group): control (DMSO was dissolved in PBS and administered once daily by i.p. injection), DHA (10 mg/kg, administered once daily by i.p. injection), Apo2L/TRAIL (50 µg, administered once daily by i.p. injection), and DHA+Apo2L/TRAIL, administered following the same schedule as the individual drugs. Tumor size was measured twice per week using calipers, and tumor volumes were estimated according to the formula: π/6×A^2^×B, where A represents the short axis, and B represents the long axis. The mice were closely monitored for 21 days prior to euthanization and removal of the tumors. Each tumor was stored at −80°C for further analysis.

### Quantitation of PCNA proliferation index

The methodology has been described previously [9]. Briefly, tumor sections were immunostained with anti-PCNA antibody. Proliferating cells were quantified by counting the PCNA positive (brown) cells and the total number of cells in 10 arbitrarily selected fields at ×400 magnification by an independent observer. The PCNA proliferation index was calculated as: the number of PCNA positive cells/the total cell count ×100%.

### *In situ* detection of apoptotic cells

The methodology has been described previously [9]. Tumor sections were stained with TUNEL agent (Roche, Shanghai, China). The apoptosis was evaluated by counting the positive cells (brown-stained), as well as the total number of cells in 10 arbitrarily selected fields at ×400 magnification by an independent observer. The apoptotic index was calculated as: the number of apoptotic cells/total number of nucleated cells ×100%.

### Statistical analysis

Tumor growth patterns were compared using the analysis of variance (ANOVA) test. Other results were expressed as the mean values ± standard deviations, and a Student's *t* test was used to evaluate statistical significance. A P value of less than 0.05 was determined to be statistically significant.
