# Correction: Dihydroartemisinin Enhances Apo2L/TRAIL-Mediated Apoptosis in Pancreatic Cancer Cells via ROS-Mediated Up-Regulation of Death Receptor 5

**DOI:** 10.1371/annotation/f7203563-87dc-4d11-a1b7-958f81cf743a

**Published:** 2012-10-11

**Authors:** Rui Kong, Guang Jia, Zhuo-xin Cheng, Yong-wei Wang, Ming Mu, Shuang-jia Wang, Shang-ha Pan, Yue Gao, Hong-chi Jiang, De-li Dong, Bei Sun

The following information was missing from the funding statement: This work was also supported in part by grants from the National Natural Scientific Foundation of China (81101799, 81170431).

